# Targeted cancer immunotherapy via combination of designer bispecific antibody and novel gene-engineered T cells

**DOI:** 10.1186/s12967-014-0347-2

**Published:** 2014-12-13

**Authors:** Katarzyna Urbanska, Rachel C Lynn, Caitlin Stashwick, Archana Thakur, Lawrence G Lum, Daniel J Powell

**Affiliations:** Department of Pathology and Laboratory Medicine, Abramson Cancer Center, Perelman School of Medicine, University of Pennsylvania, 3400 Civic Center Blvd, Bldg 421, Smilow CTR, Rm 08-103, Philadelphia, PA 19104-5156 USA; Department of Obstetrics and Gynecology, Ovarian Cancer Research Center, Perelman School of Medicine, University of Pennsylvania, Philadelphia, PA USA; Department of Oncology, Wayne State University, Detroit, MI USA

**Keywords:** Immunotherapy, Adoptive T cell transfer, Chimeric immunoreceptor, Cancer, Bispecific antibody, Trastuzumab, Rituximab

## Abstract

**Background:**

Redirection of T lymphocytes against tumor antigens can induce dramatic regression of advanced stage malignancy. The use of bispecific antibodies (BsAbs) that bind both the T-cell receptor (TCR) and a target antigen is one promising approach to T-cell redirection. However, BsAbs indiscriminately bind all CD3+ T-cells and trigger TCR activation in the absence of parallel costimulatory signals required to overcome T-cell unresponsiveness or anergy.

**Methods:**

To address these limitations, a combination platform was designed wherein a unique BsAb referred to as frBsAb exclusively engages T-cells engineered to express a novel chimeric receptor comprised of extracellular folate receptor fused to intracellular TCR and CD28 costimulatory signaling domains in tandem; a BsAb-binding immune receptor (BsAb-IR). As a surrogate TCR, the BsAb-IR allows for concomitant TCR and costimulatory signaling exclusively in transduced T-cells upon engagement with specific frBsAbs, and can therefore redirect T-cells on command to desired antigen. Human primary T-cells were transduced with lentiviral vector and expanded for 14–18 days. BsAb-IRs were harvested and armed with frBsAbs to test for redirected cytotoxicity against CD20 positive cancer cell lines.

**Results:**

Using frBsAbs specific for CD20 or HER2, the lytic activity of primary human T-cells expressing the BsAb-IR was specifically redirected against CD20+ leukemic cells or HER2+ epithelial cancer cells, respectively, while non-engineered T-cells were not activated. Notably, elimination of the CD28 costimulatory domain from the BsAb-IR construct significantly reduced frBsAb-redirected antitumor responses, confirming that frBsAbs are capable of delivering simultaneous TCR activation and costimulatory signals to BsAb-IR T-cells.

**Conclusion:**

In summary, our results establish the proof of concept that the combination of BsAbs with optimized gene-engineered T-cells provides the opportunity to specify and augment tumor antigen-specific T-cell activation and may improve upon the early success of conventional BsAbs in cancer immunotherapy.

**Electronic supplementary material:**

The online version of this article (doi:10.1186/s12967-014-0347-2) contains supplementary material, which is available to authorized users.

## Introduction

Antigen-specific monoclonal antibodies (mAbs) are established as immunotherapeutic agents for the treatment of human malignancies such as non-Hodgkin lymphoma (NHL), CD30-positive lymphoma [1,2], EGFR-expressing advanced bowel cancer, metastatic colorectal carcinoma [3-6]. However, the therapeutic efficacy of tumor antigen-specific mAbs can be limited in cancer therapy due to their poor recruitment of the adaptive immune response. To address this, other strategies were employed, including the development of bispecific antibodies (BsAbs) [7,8]. While a mAb recognizes a single antigen target and closely resembles a naturally-occurring antibody, a BsAb is a synthetic construct that aligns two antigen-specific binding potentials within one molecule enabling the linking of two distinct antigens [9]. BsAbs generally couple T-cells, through a T-cell receptor (TCR)-CD3-specific antibody, with target cells, via an antigen-specific antibody. As a result, cancer cells are killed when cytotoxic T lymphocytes are engaged to antigen-expressing tumor cells and simultaneously activated by the arm of the BsAb that triggers TCR activation [10,11]. Most BsAbs rely on re-direction of cytotoxic T-cells, the most powerful effector cells of the immune system [12], where the BsAb indiscriminately engages all available TCR CD3 molecules and overrides the natural antigen-specificity of T-cells.

While TCR activation alone by BsAbs can activate T-cells, stimulation of T-cell activity is a complex, sophisticated process regulated by an assortment of molecules that provide activating, inhibiting or costimulatory signals to T-cells. One fundamental tenet of T-cell immunobiology is that sustained stimulation via TCR CD3 (signal 1) without parallel costimulatory signals, such as those provided by CD28 receptor, results in impaired T-cell activation with induction of anergy or apoptosis [13]. Accordingly, CD3-based immunotherapy with BsAbs may be improved by provision of accessory costimulation *ex vivo* or *in vivo* to elicit potent, long-lasting antitumoral effects. This can be achieved by *ex vivo* activation of cytotoxic T-cells [14,15], or by systemic administration of IL-2 cytokine [16,17]. Alternatively, technological advances have led to the development of new BsAb strategies which simultaneously trigger the activation of costimulatory receptors (e.g., CD28, 4-1BB, OX40) in conjugation with conventional BsAbs treatment [18,19]. Parallel costimulatory signaling can also be provided by combining BsAbs with an agonistic anti-CD28 mAb to mediate a synergistic effect in eliciting an antitumor response *in vitro* [20,21]. Similarly, 4-1BB-mediated costimulation at the tumor site can enhance T-cell activation mediated by a BsAb [22,23], as evidenced by increased T-cell cytokine release, activation marker expression, and proliferation. While it is increasingly evident that BsAb approaches that incorporate parallel costimulation are more effective than conventional BsAb, the undefined optimal stoichiometry of multiple receptor engagement and the indiscriminant nature of T-cell engagement represent still represent challenges to the field.

Here, we sought to establish a proof of concept that the needs for costimulation, fixed stoichiometry and T-cell specification of conventional BsAbs can be resolved through the use of advanced T-cell engineering strategies. We and others have previously shown that human T-cells engineered to express a chimeric antigen receptor (CAR) containing an extracellular tumor antigen-specific antibody fused to intracellular TCR CD3 and costimulatory domains in tandem receive dual TCR (signal 1) and costimulatory (signal 2) upon antigen encounter that reinforce T-cell activation, proliferation and cancer killing [24-26]. Based upon this principle, we have designed a novel platform that combines the application of a BsAb with T-cells that are genetically engineered to express a unique BsAb-binding immune receptor (BsAb-IR). Here, the BsAb-IR is comprised of a portion of an extracellular folate receptor (FR; 231aa) fused to intracellular TCR and CD28 costimulatory signaling domains in tandem, and can be bound and activated by an anti-FR antibody arm of a unique BsAb that bridges FR and tumor antigen (frBsAb). Using frBsAbs of diverse antigen specificities, we show that tumor antigen-specific frBsAbs specifically bind target antigen on human tumor cells and, upon co-engagement of the BsAb-IR on engineered T-cells, delivers simultaneous TCR CD3 activation and CD28 costimulation signals in a target dependent manner, resulting in the selective augmentation of activation, proliferation and antitumor activity of BsAb-IR T-cell subset.

## Materials and methods

### BsAb-binding immune receptor (BsAb-IR) construction

Folate Receptor alpha (FR) DNA sequence was amplified using primers: 5′-AAAAGCCTAGGATCC-3′ and 5′-AACCGCGCTAGCAAA-3′. After amplification and the insertion of 3′-Bam-H1 and 5′-Nhe-1 restriction sites, PCR product was digested with Bam-HI and NheI enzymes and ligated into pELNS, a third generation self-inactivating lentiviral expression vector, containing human CD3z or CD28-CD3z signaling endodomains, under an EF-1α promoter. The resulting constructs were designated pELNS FBIR-zeta and pELNS FBIR-28z, respectively.

### Recombinant lentivirus production

High-titer replication-defective lentiviral vectors were produced and concentrated as previously described [27,28]. Briefly, 293 T human embryonic kidney cells were transfected with pVSV-G (VSV glycoprotein expression plasmid), pRSV.REV (Rev expression plasmid), pMDLg/p.RRE (Gag/Pol expression plasmid), and pELNS transfer plasmid using Lipofectamine 2000 (Invitrogen). The viral supernatant was harvested at 24 and 48 h post-transfection. Viral particles were concentrated and resuspended in 0.5 ml by ultracentrifugation for 2.5 h at 25,000 rpm with a Beckman SW28 rotor (Beckman Coulter, Fullerton, CA).

### T-cells

Primary human CD4+ and CD8+ T-cells were isolated from healthy volunteer donors following leukapheresis by negative selection, and purchased from the Human Immunology Core at University of Pennsylvania. All specimens were collected under a University Institutional Review Board-approved protocol, and written informed consent was obtained from each donor. T-cells were cultured in complete media (RPMI 1640 supplemented with 10% heat inactivated fetal bovine serum (FBS), 100 U/ml penicillin, 100 ug/ml streptomycin sulfate, 10-mM HEPES), and stimulated with anti-CD3 and anti-CD28 mAbs coated beads (Invitrogen) as described. 24 hr after activation, T-cells were transduced with lentiviral vectors at MOI of ~5-10. Human recombinant interleukin-2 (IL-2; Novartis) was added every other day to 50 IU/ml final concentration and a 0.5-1×10^6^ cells/ml cell density was maintained. Rested engineered T-cells were adjusted for identical transgene expression prior to functional assays. For the investigation into the effects of dose of folic acid, T-cells were cultured in RPMI 1640, 10% heat inactivated fetal bovine serum (FBS), 100 U/ml penicillin, 100 ug/ml streptomycin sulfate, 10-mM HEPES) supplemented with 40 mg/l folic acid (Sigma-Aldrich).

### Cell lines

Lentivirus packaging was performed in the immortalized normal fetal renal 293 T cell line purchased from ATCC. Human cell lines used in immune based assays include CD20 positive cell lines Ramos and Daudi. 293 T cells and tumor cell lines were maintained in RPMI-1640 (Invitrogen) supplemented with 10% (v/v) heat-inactivated FBS, 2 mM L-glutamine, and 100 μg/mL penicillin and 100U/mL streptomycin. All cell lines were purchased from ATCC.

### BsAb heteroconjugation

The BsAbs were engineered by heteroconjugating cross-linked mAbs. Anti-CD3 (Orthoclone OKT-3, Orthobiotech, Bridgewater, NJ, USA) or anti-FR alpha (MOV18 Ab, Enzo Life Sciences) was cross-linked with 2-iminothiolane HCl (Pierce, Rockford, IL, USA) or trastuzumab (Herceptin, Genentech, San Francisco, CA) and rituximab (Rituxan; Genentech, South San Francisco, CA, USA) was cross-linked with sulphosuccinimidyl 4-(N-maleimidomethyl) cyclohexane-1-carboxylate (Pierce), purified on PD-10 columns (BioRad Laboratories, Hercules, CA, USA), and heteroconjugated overnight at 4°C and analyzed by non-reducing SDS-PAGE as described earlier [29,30].

### Flow cytometric analysis

The following mAbs were used for phenotypic analysis: APC-Cy7 Mouse Anti-Human CD3; FITC-anti-human CD4; APC-anti-human CD8; (BD Biosciences). Tumor cell surface expression of HER2 was detected by trastuzumab antibody (Herceptin, Genentech, San Francisco, CA), CD20 by biotinylated rituximab (Rituxan; Genentech, South San Francisco, CA, USA) followed by incubation with Strepavidin-APC. BsAb-IR expression was detected by Mov18/ZEL antibody (Enzo Life Sciences) followed by anti-mouse FITC or APC conjugated antibody (LifeBioscience). The isotype controls mIgG-APC-Cy7, mIgG-FIT, mIgG-APC, mIgG-PerCpCy5, mIgG-Pacific-Blue-A, and mIgG-PE-Cy7 were from eBiosciences and anti-mouse-AF488 from Invitrogen. Flow cytometric data were analyzed by FlowJo software. For intracellular staining, cells were permeablized using the FoxP3 staining buffer set (eBioscience), according to manufacturer’s instructions and labeled with: anti-human IFNg-PacificBlue, anti-human IFNg-PerCpCy5, anti-human TNFa-APC, anti-human IL2-PeCy7 (BD Biosciences).

### Cytokine release assays and intracellular cytokine staining

Cytokine release assays were performed by co-culture of 1×10^5^ BsAb-IR T-cells with immobilized MOV18Ab (anti-FR alpha) or IgG1 (100 ng/ml). For co-culture experiments against tumor cells, 1×10^5^ target cells were labeled with frBsAbs at 100 ng/10^6^ cells for 30 min at 4°C, per well in triplicate in 96-well round bottom plates, T-cells were added into the culture at the E:T 1:1 ratio, in a final volume of 200ul of T-cell media. After 16 h, co-culture supernatants were assayed for presence of IFNg using an ELISA Kit, according to manufacturer’s instructions (Biolegend). Values represent the mean of triplicate wells. IFNg, IL2, IL-4, TNFa and MIP-1a cytokines were measured by flow cytometry using Cytokine Bead Array, according to manufacturer’s instructions (BD Biosciences). For cytokine staining cells were stimulated in 37°C for around 6 h in the presence of Golgistop (BD Biosciences) and monensin (Sigma). Cells were washed, Fc blocked, and stained with MOV18AB-FITC then permeablized and stained with anti-IFNg, TNFa, or isotype controls in permeablization buffer for 30 min.

### Cytotoxicity assays

^51^Cr release assays were performed as described [31]. Target cells were labeled with frBsAbs at 100 ng per 10^6^ cells for 30 min at 37°C in PBS/2%FBS. Next, antibody labeled cells were labeled with 100uCi 100 mCi ^51^Cr at 37°C for 1.5 hours. Target cells were washed three times in PBS, resuspended in CM at 10^5^ viable cells/mL and 100uL added per well of a 96-well V-bottom plate. Effector cells were washed twice in CM and added to wells at the given ratios. Plates were quickly centrifuged to settle cells, and incubated at 37°C in a 5% CO_2_ incubator for 4 hours. The supernatants were harvested, transferred to a lumar-plate (Packard) and counted using a 1450 Microbeta Liquid Scintillation Counter (Perkin-Elmer). Spontaneous ^51^Cr release was evaluated in target cells incubated with medium alone. Maximal ^51^Cr release was measured in target cells incubated with SDS at a final concentration of 2% (v/v). Percent specific lysis was calculated as (experimental - spontaneous lysis/maximal - spontaneous lysis) times 100.

### Statistical analysis

Student’s *t* test was used to evaluate differences in T-cells specific cytolysis. GraphPad Prism 4.0 (GraphPad Software) was used for the statistical calculations. *P* < 0.05 was considered significant.

## Results

### Construction of a novel bispecific antibody platform

**Figure 1:**
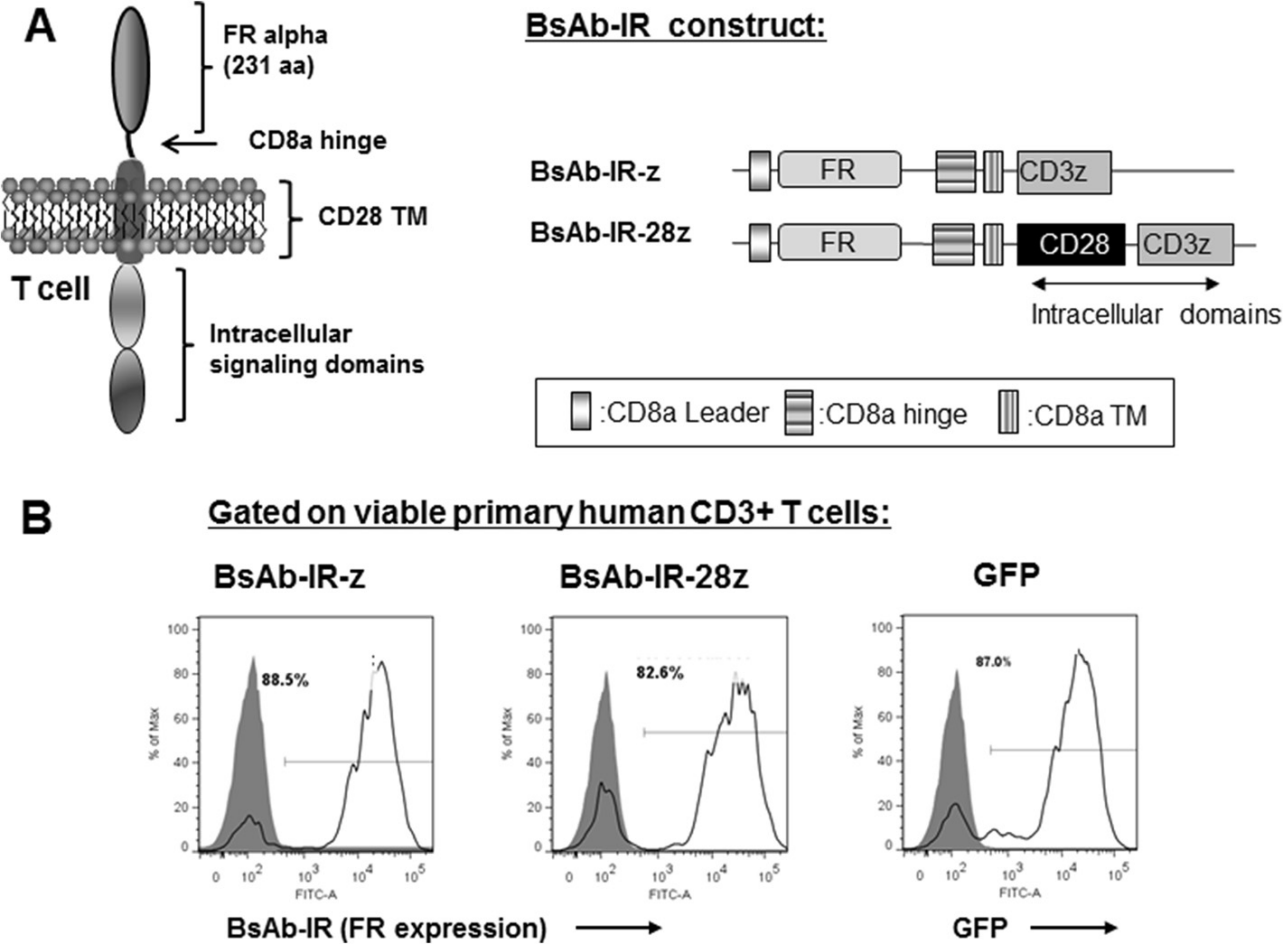
**Generation of primary human T cells expressing costimulated ligand for redirectionagainst tumor antigens via bispecific antibody. A**. *Upper left* Schematic representing the costimulated ligand; BsAb-IR. *Upper right* Schematic representation of Folate Receptor alpha based BsAB-Immune Receptor gene constructs containing extracellular portion of FRa (231aa) fused to the human CD3z cytosolic domain alone (BsAb-IR-z) or in combination with the CD28 co-stimulatory module (BsAb-IRFBIR-28z). **B**. BsAb-IR transgene expression (open histograms) was detected by Mov18/ZEL antibody (Enzo Life Sciences) followed by anti-mouse FITC or and via GFP expression for control, GFP transduced T-cells. Staining was performed 5 days following transduction with lentivirus and compared to untransduced T cells, or isotype stained T cells (grey filled histograms). Percent transduction is indicated.

We sought to design and construct a combination platform that merges advantages of BsAb technology with that of gene-engineered T-cell therapy. Based upon its limited endogenous expression by human T-cells, folate receptor alpha (FR) was selected for use in immunoreceptor construction. We constructed a folate binding immunoreceptor construct comprised of the extracellular portion of FR (1-231aa) linked to a CD8α hinge and transmembrane region, followed by a CD3z signaling moiety alone (BsAb-IR-z; Figure 1A Schematic). In order to incorporate simultaneous CD28-mediated signaling and evaluate whether a functional secondary signal potentiates T-cell activity, a second-generation BsAB-IR that includes a chimeric fusion of CD28 and CD3-z intracellular domains was constructed (BsAb-IR-28z). GFP transduced, or non-transduced T-cells were used as a specificity control in all experiments. BsAb-IR-z and BsAb-IR-28z immunoreceptor sequences were transduced into freshly isolated human primary T-cells utilizing lentiviral vectors. Five days following lentiviral transduction, BsAb-IR cell surface expression of was measured via detection of surface FR expression by flow cytometric analysis utilizing an anti-FRa antibody (MOV18) with a FITC-labeled anti-mouse secondary antibody. As shown in Figure 1B, transduction efficiencies were >80% for all constructs. For all functional assays, transduced T-cells were equilibrated to 70% by adding untransduced T-cells. In all T-cell cultures the ratio of CD4 and CD8 T cells was approximately 30% CD4 and 70% CD8 (data not shown).

### Heteroconjugation of BsAb-IR bispecific antibodies

**Figure 2:**
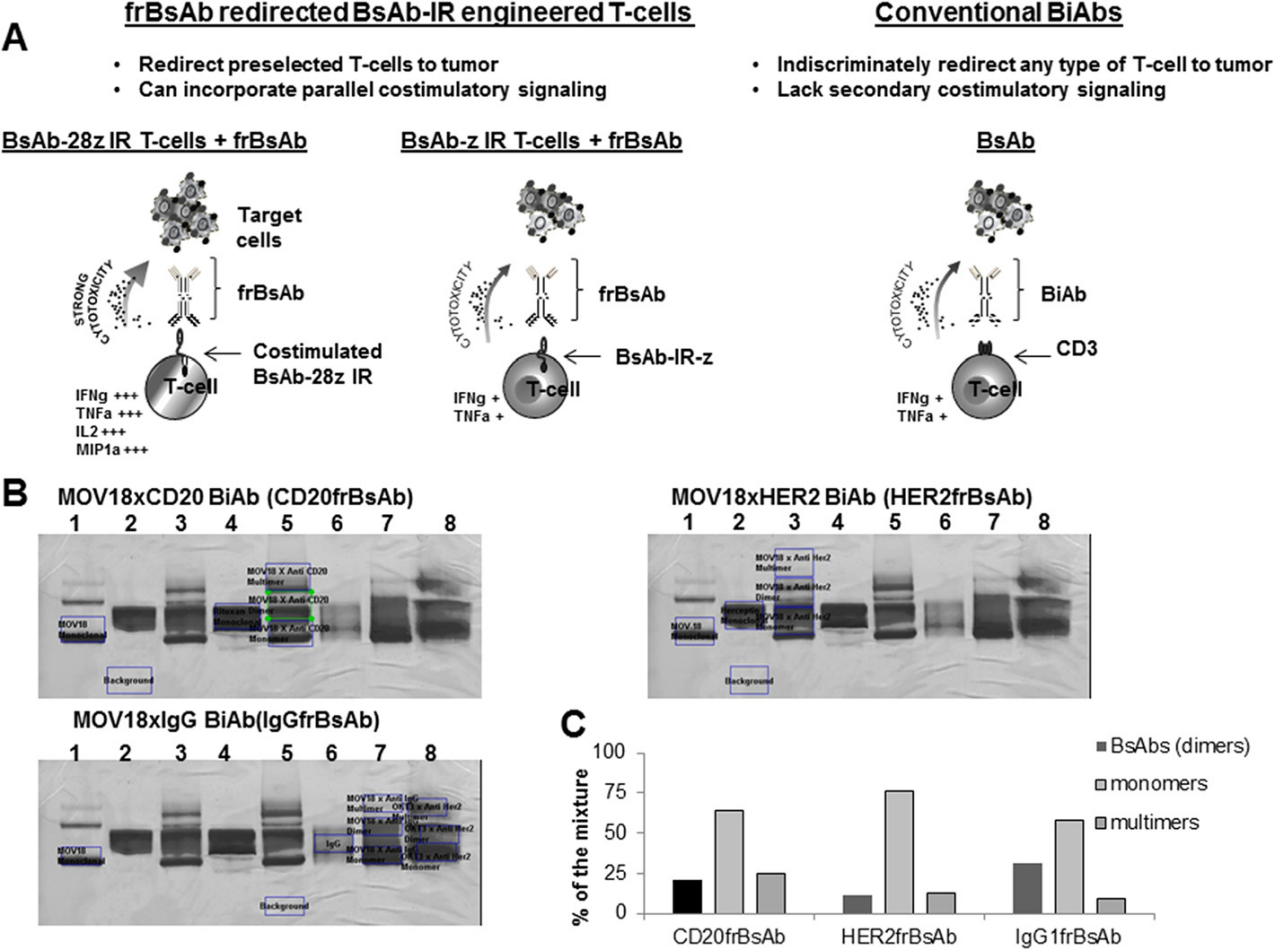
**A. Schematic illustration of the functional activity of primary human T-cells engineered to express a chimeric bispecific antibody binding immune receptor (BsAb-IR) when redirected against tumor cells with a unique bispecific antibody (frBsAbs).** The frBsAb, bound to tumor associated antigen (TAA) on tumor cell surface, facilitates crosslinking with BsAb-IRs on the T-cell surface and promotes BsAb-IR T-cell activation. Unlike traditional bispecific antibody (BsAbs) platforms that indiscriminately engage all T-cells via the CD3 molecule and lack costimulatory signaling capacity, incorporation of CD28 co-stimulation in a second generation BsAb-28z-IR augments antitumor activity of redirected human T-cells, in comparison to a first generation BsAb-z-IR lacking an engineered costimulatory domain. In addition, engineered expression of BsAb-IR in preselected human T cells ex vivo allows for a selective activation of the chosen T-cell population, not the open repertoire of all T cells. **B**. SDS-PAGE (4-15% gel) analysis of chemically heteroconjugated anti-CD3 [OKT3 (immunoglobulin (Ig)G2a); Orthobiotech], or MOV18 (anti-FR alpha) with anti-Her2/neu (Herceptin, Genentech, San Francisco, CA) or anti-CD20 (Rituxan, Genentech) and control IgG. The SDS-polyacrylamide gel electrophoresis shows anti-BsAb-IR monoclonal antibody, MOV18 (lane 2); anti-CD20 monoclonal antibody, (lane 4); and the heteroconjugated product containing monomers, dimers, and multimers (lane 3, 5 and 7 and 8). **C**. Graph showing the percentage of heterodimerized bispecific antibody in the mixture for each antibody format following chemical conjugation.

The BsAb-IR engagers, referred to as frBsAbs, were produced by chemical heteroconjugation of two mAbs (Additional file 1: Figure S1), one that binds the extracellular FR portion of the BsAb-IR (MOV18Ab) on engineered T-cells and another that targets a chosen tumor-associated antigen (TAA) (Figure 2A). mAb against CD20 molecule (rituximab) was used for tumor targeting. Antibody recognizing HER2 (trastuzumab) and an isotype IgG1 were used as specificity controls in the construction of frBsAbs. Figure 2B shows Western Blot analysis of the cross-linked mAbs, following overnight heteroconjugation at 4°C. The products of the heteroconjugation were resolved by SDS-PAGE (4–15% gradient) and stained with Gelcode Blue (Pierce). By densitometric quantitation using Gel Doc analysis and Quantity One software (Bio-Rad), the mixture consisted of 21% of the desired conjugated heterodimers, 64% unconjugated monomers and 25% multimers for the CD20-frBsAb preparation. The HER2frBsAb mixture consisted of 11% conjugated heterodimers, 76% monomers and 13% multimers, and the IgGfrBsAb control mixture was 31%, 58%, and 9%, respectively as represented on the graph (Figure 2C). Previous preclinical and clinical studies have shown no significant difference in binding and specificity between the purified and unpurified heteroconjugates [29]. Therefore, the unpurified heteroconjugated material was used for arming of the BsAb-IR engineered T-cells.

### Primary human T-cells expressing BsAb-IR are specifically activated by immobilized anti-BsAb-IR antibody

**Figure 3:**
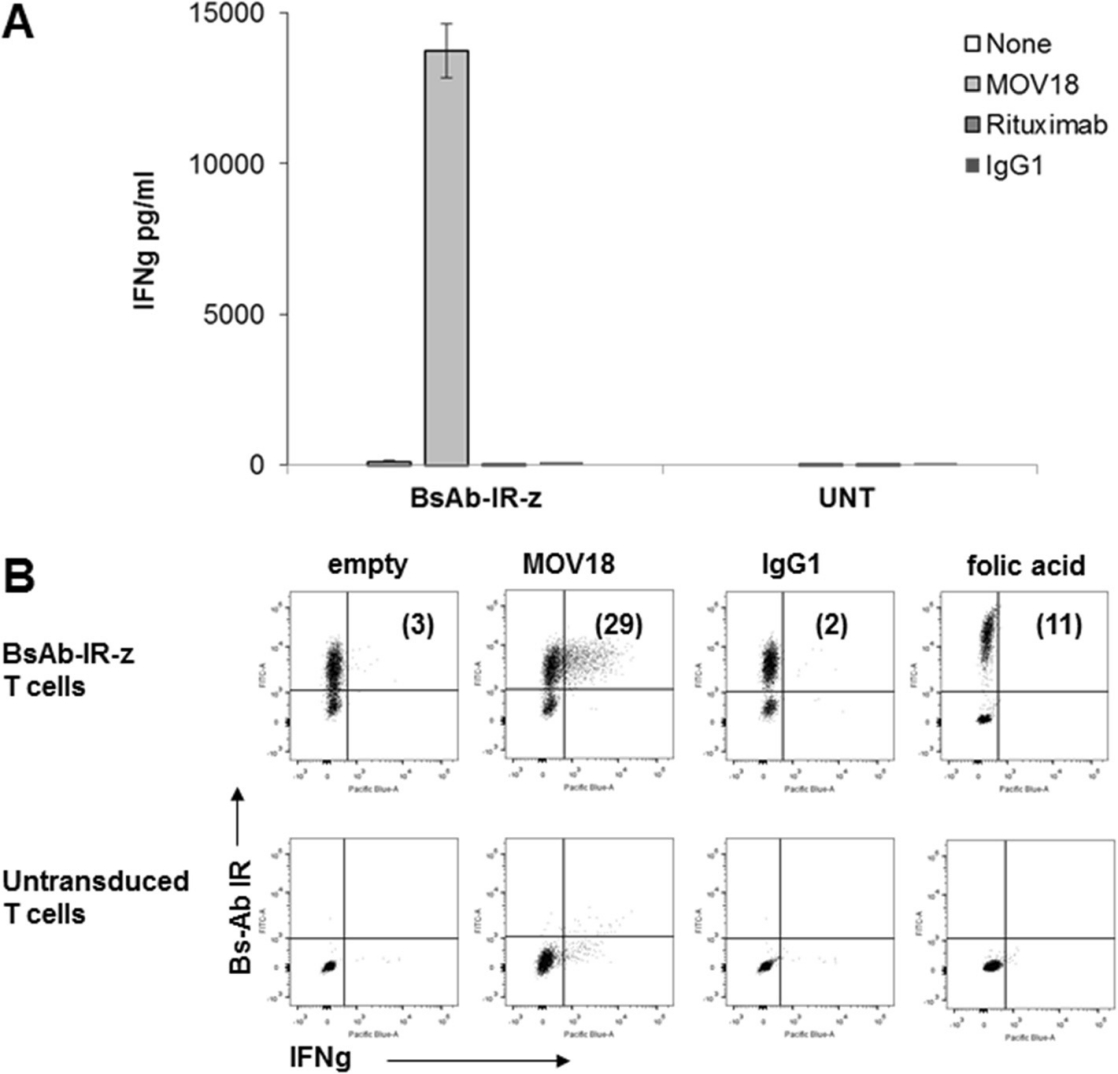
**Activation of BsAb-IR expressing T-cells depends on the specific engagement and crosslinking of immunoreceptors. A**. IFNg production by BsAb-IR-z and untransduced T-cells stimulated with immobilized anti-BsAB-IR antibody (MOV18), anti-CD20 rutixumab, or mouse IgG1. Overnight culture supernatants were analyzed for human IFNg cytokine by ELISA. Concentration of IFNg is expressed as mean ± SEM in pg/ml from triplicate wells. **B**. Intracellular IFNg staining of BsAb-IR-z and untransduced T-cells following 6 hr stimulation with 0.1ug of an immobilized anti-BsAb-IR (MOV18) or IgG1 antibody, or cultured in a growth media supplemented with folic acid (40 mg/l) for 12 hr.

To determine whether crosslinking of BsAB-IRs on the T-cell surface results in a specific immune-activation of engineered T-cells, BsAb-IR T-cells were cultured in plates coated with immobilized anti-BsAb-IR antibody (MOV18 Ab), control IgG1 or rituximab, or left uncoated. BsAb-IR-z expressing T-cells exclusively produced pro-inflammatory cytokine interferon-g (IFNg) in response to anti-BsAb-IR antibody (MOV18 Ab) stimulation, but not to control antibodies; anti-CD20 (rutixumab) and IgG1 (Figure 3A). Importantly, untransduced T cells did not show any significant immune-reactivity. Single cell analysis by intracellular cytokine production confirmed that IFNg production was restricted to T cells expressing BsAb-IR-z; T cells lacking BsAb-IR did not (Figure 3B). Since the BsAb-IR comprise extracellular portion FRa which binds folic acid with high affinity, we tested whether high levels of folic acid can mediate BsAb-IR T-cell activation. As shown by intracellular cytokine analysis, BsAb-IR T-cells did not produce IFNg in the presence of supra-physiological levels of folic acid.

### Costimulatory properties of BsAb-binding immune receptor (BsAb-IR)

**Figure 4:**
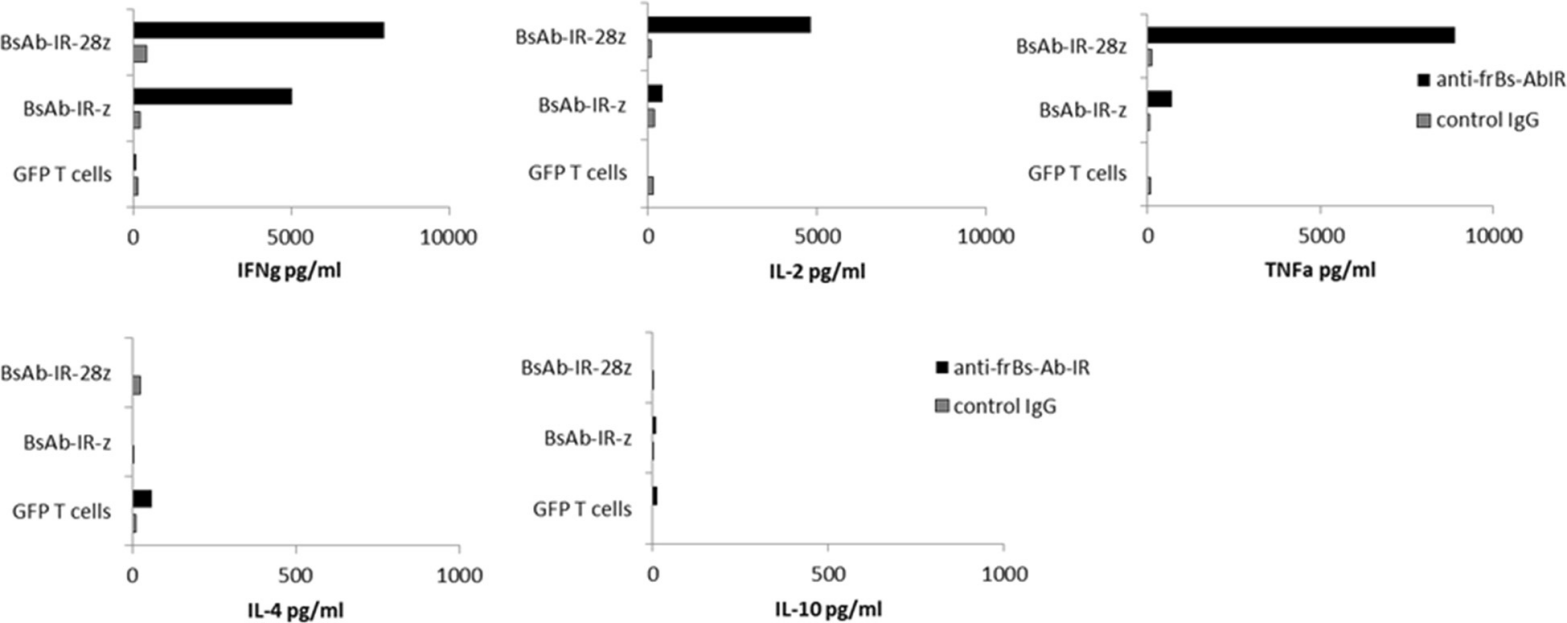
**The amount of cytokines produced by BsAb-IR+ T-cells is dependent on the presence of the CD28 costimulatory domains within the immune receptor construct.** Comparison of cytokine production by T-cells transduced with different BsAb-IR constructs. Pooled supernatant from triplicate co-cultures were measured for proinflammatory cytokine secretion by cytokine bead assay. IFNg, IL-2, TNFa, IL-4 and IL-10 cytokine concentrations (pg/ml) were measured from cultures of BsAb-IR-z, BsAb-IR-28z and control GFP transduced T-cells stimulated with 0.1ug of an immobilized anti-BsAb-IR (MOV18) antibody. Representative data from 4 independent assays are shown.

Production of multiple proinflammatory cytokines can improve cytolytic function and contribute to in vivo activity [32]. Costimulation promotes T-cell activation, proliferation and effector function and therefore represents a key aspect in potentiating T-cell responses in cancer immunotherapy. The costimulatory properties of BsAB-IR were therefore evaluated in an experimental setting where the first signal required for T-cell activation and the second costimulatory signal are triggered by the engagement of BsAb-IR-28z by frBsAb. CD28 is one of the best-characterized costimulatory molecules expressed by T-cells, providing signals critical for optimal T-cell activation, cytokine production, proliferation, and survival [33]. Having incorporated the CD28 endodomain into the BsAB-IR, we evaluated the contribution of CD28 costimulatory signals in BsAB-induced activation of human T-cells. We first tested whether cross-linking of the costimulated BsAb-IR-28z receptor induces more potent T-cell activity in comparison to activation triggered by the first generation BsAb-IR-z immunoreceptor, which lacks a costimulatory signaling domain. BsAb-IR-28z, BsAb-IR-z or untransduced T-cells were added to wells containing immobilized anti-FR antibody (MOV18) or IgG isotype antibody, and incubated for 16 hours before culture supernatants were collected and measured for type and amount of proinflammatory cytokines secreted in response to specific stimulation. BsAb-IR-28z and BsAb-IR-z T-cells both secreted IFNg, interleukin-2 (IL-2) and tumor necrosis factor-alpha (TNFa) cytokines when stimulated with anti-FR, but not control, antibody (Figure 4). Little to no IL-4 or IL-10 cytokines was detected suggesting a preferential Th1-cytokine response. GFP transduced control T-cells did not secrete cytokine under either antibody condition. More notably, primary human T-cells expressing a BsAb-IR containing the CD28 endodomain secreted higher levels of proinflammatory cytokines compared to T-cells with the BsAb-IR-z alone (n = 4). Similar increases in the levels of secreted IFNg, IL-2 and TNFa cytokines occurred in the BsAb-IR-28z group. In all cases, cytokine secretion by BsAb-IR T-cells was specific, and dependent on the engagement of the BsAb-IR, demonstrated by little constitutive cytokine production in the absence of immobilized anti-FR antibody, or in the presence of the isotope control antibody. Importantly, stimulation of BsAbIR-T cells with immobilized antibodies including: trastuzumab, rituximab and/or IgG1 did not induce secretion of proinflamatory cytokines (data not shown).

### Evaluating BsAb-directed antitumor responses of BsAb-IR+ T-cells *in vitro*

**Figure 5:**
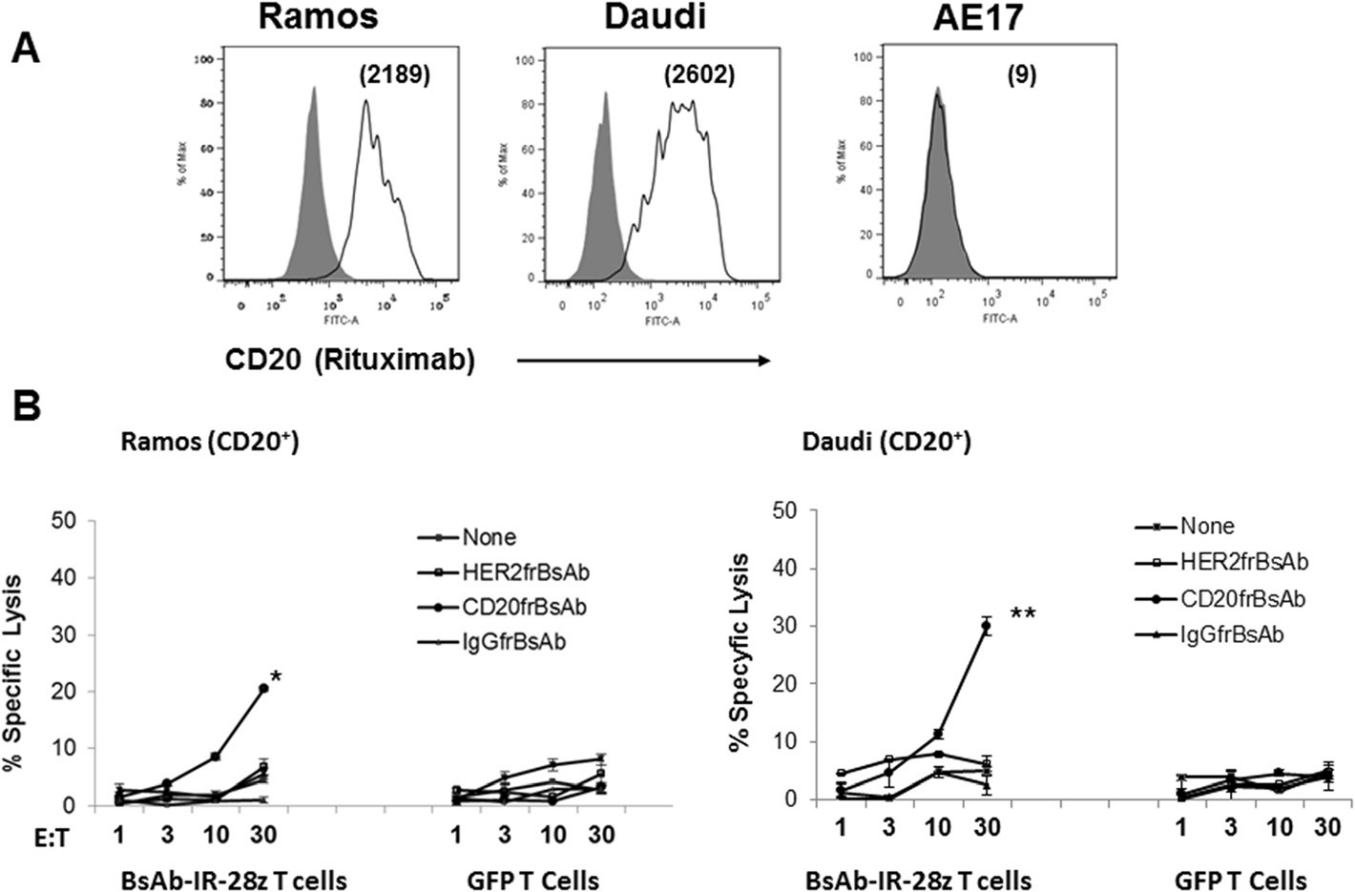
**frBsAb-mediated activation of T cells by antigen-bound bispecific antibody (frBsAb) facilitates specific target cell killing. A**. The CD20 cell surface expression (open histograms) on cancer cell lines was detected with biotinylated anti-CD20 mAb rituximab and evaluated by flow cytometry. Numbers represent mean fluorescence intensity (MFI). **B**. Antigen-specific tumor killing by CD20frBsAb redirected BsAb-IR T-cells. Primary human T-cells transduced to express BsAb-IR-28z or GFP (control) were co-cultured with Cr^51^-labeled Ramos, and Daudi tumor cells, pretargeted with indicated frBsAb for 4 hrs at the indicated effector to target ratio. Percent specific target cell lysis was calculated as (experimental - spontaneous release) ÷ (maximal - spontaneous release) × 100. Data represent the means ± SD for 3 different experiments. * P = 0.0082 for BsAb-IR-28z T-cells/CD20frBsAB versus IgGfrBsAb redirected BsAb-IRs against Ramos cells. ** P = 0.0166 for BsAb-IR-28z T-cells/CD20frBsAB versus IgGfrBsAb redirected BsAb-IRs against Daudi cells.

Finally, we investigated the cytotoxic potential of BsAb-IR T-cells. To evaluate whether BsAb-IR-mediated activation of T-cells by cancer cell bound frBsAb facilitates specific target cell killing, BsAb-IRs T cells were assayed for their capacity to specific lyse Ramos, Daudi and AE17 cell lines. We first evaluated the potency of the combination of a CD20frBsAb and BsAb-IR T-cells. Cell surface expression of CD20 on lymphoma cell targets was confirmed by flow cytometry. Human Burkitt’s lymphoma cell lines, Ramos and Daudi, expressed high levels of CD20, with a specific mean fluorescence intensity (MFI) of 2189 and 2602 respectively (Figure 5A). The mouse mesothelioma cell line, AE17 did not express CD20 antigen on cell surface and was used as an antigen specificity control. Target cells were exposed to frBsAb bearing either an anti-CD20 (CD20frBsAb), irrelevant anti-HER2 (HER2frBsAb) or control IgG (IgGfrBsAb) antibody arm at 100 ng per 10^6^ cells for 30 minutes, labeled with ^51^Cr, and washed before culture with T-cells. Cytotoxicity was measured at indicated E:T ratios of 1, 3, 10 and 30 to 1 in standard 4-hour ^51^Cr-release assays. BsAb-IR T-cells exhibited specific redirected cytotoxic activity against CD20+ lymphoma cells when using CD20-frBsAbs at E:T ratios >10:1. Cytotoxic activity was not detected in co-cultures where anti-HER2 or IgG control frBsAbs were used for redirection (Figure 5B). After 4 hours, BsAb-IR transduced T-cells specifically lysed CD20frBsAbs targeted Ramos (20% ± 3%, 30:1) and Daudi (30% ± 4%, 30:1) cell targets (P < 0.05), but not when redirected with control HER2 or IgG1frBsAbs (5% ± 1.5% and 4.6% ± 2%, respectively at 30:1; n = 3). CD20frBsAb redirected cytotoxicity of the CD20-negative AE17 cell line was not observed at any E:T ratio (Additional file 2: Figure S2).

**Figure 6:**
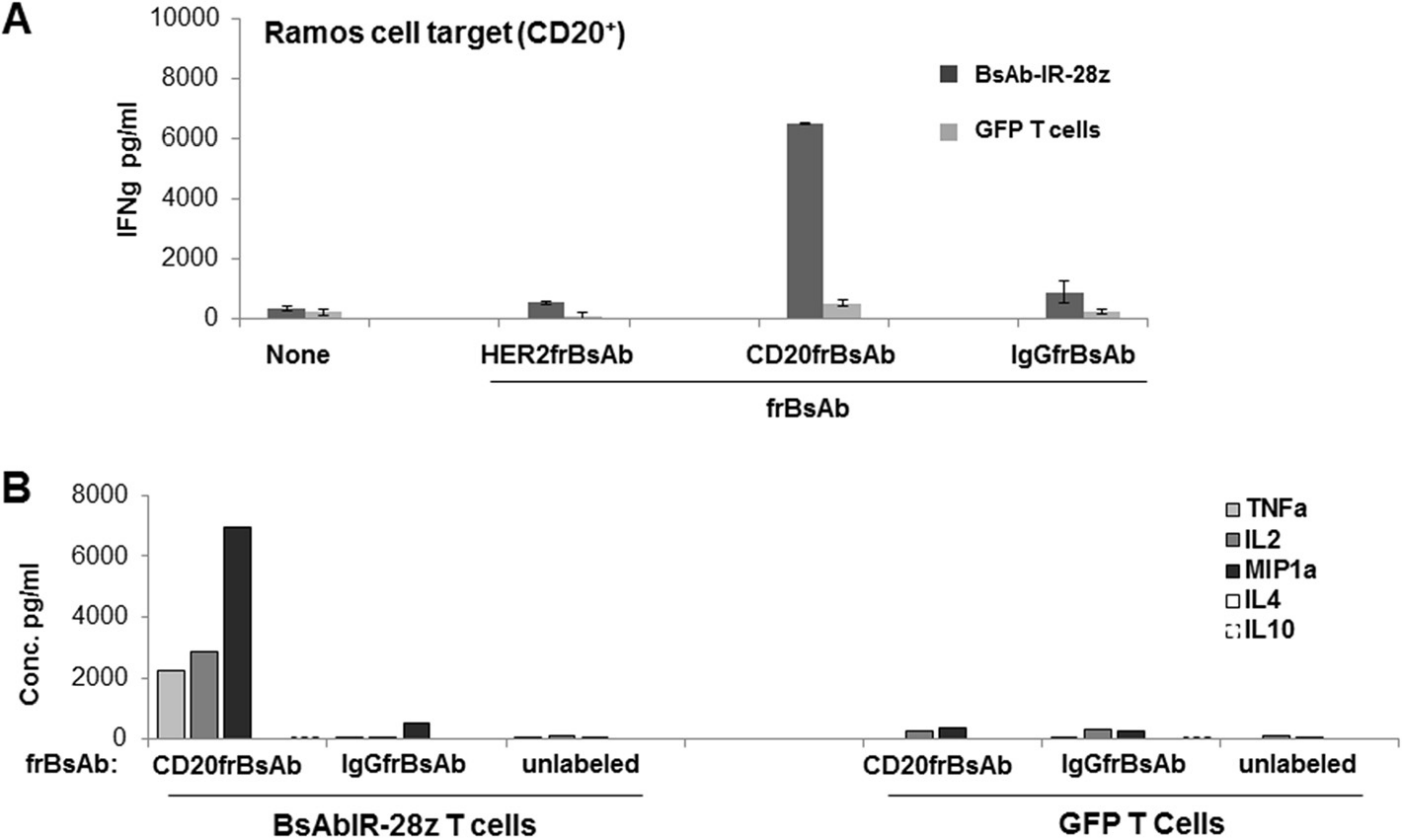
**Anti-tumor activity of BsAb-IR-28z engineered T-cells. A**. BsAb-IR-28z + T lymphocytes produce inflammatory cytokines when redirected with antigen-bound frBsAbs. CD20 redirected BsAb-IR, but not GFP (control) T-cells produce high levels of IFNg against CD20-positive Ramos tumor cells. BsAb-IR-28z or GFP transduced T-cells were cultured overnight with an equal number of CD20frBsAb, or control frBsAbs pretargeted CD20-positive Ramos cancer cells. Overnight culture supernatants were analyzed for human IFNg cytokine by ELISA. Concentration of IFNg is expressed as mean ± SEM in pg/ml from triplicate wells. **B**. Cytokine bead-array analysis of cytokine production (pg/ml) by BsAb-IR-28z + T-cells or GFP+ T-cells. Supernatants from three independent cultures were harvested 16 h post-stimulation, pooled and assessed for measurable cytokine.

In parallel studies, BsAb-IR-28z T-cells, but not control GFP T-cells, produced high levels of IFN-g in co-culture with Ramos cells but only when redirected with a CD20frBsAb (Figure 6A). Redirection with other frBsAbs had no impact on cytokine secretion. Culture supernatants were pooled and measured for various T-cell produced cytokines by cytokine bead array assay. Similar to IFNg, IL-2, TNFa and MIP-1a cytokines were all detected in supernatants from the co-culture of BsAb-IR-28z T-cells, but not GFP T-cells, and Ramos cells exposed to CD20frBsAb (Figure 6B). IL-4 and IL-10 secretion were not detected. Again, no substantial cytokine production by BsAb-IR-28z T-cells or GFP T-cells was observed in the presence of antigen specificity control frBsAbs.

## Discussion

Tumor cells express various proteins on their surfaces that differentiate them from healthy tissues, either by levels of expression or by revealing novel antigens. Multiple antibodies against tumor associated antigens (TAAs) have been identified and exploited against different types of cancers though their mode of activity is largely independent of T-cell involvement. In order to enlist and mobilize of T-cells against cancer and maximize antibody targeted therapy, innovative methods have been designed to allow T-cells to be redirected against TAAs via TAA-specific antibodies. In one approach, relying on genetic retargeting of cytotoxic T-cells with antibody [34,35], a chimeric gene constructed that encodes an extracellular antibody specific for a TAA in the form of a single-chain variable fragment (scFv) in tandem with a gene sequence that encodes intracellular T-cell signaling domains, such as those from the CD3zeta subunit of the T cell receptor complex for T-cell activation and from costimulatory molecules to support T-cell effector function, proliferation and survival. The gene encoding this chimeric antigen receptor (CAR) is then introduced into T-cells for adoptive T-cell therapy [36]. Transfer of autologous T-cells engineered to express CARs with costimulatory domains has shown remarkable anti-tumor activity in CD19+ hematological malignancies [37,38]. Another strategy to target T-cells to epitopes recognized by antibodies is to develop dual specificity antibody-like molecules that possess antigen-binding specificity for both a protein expressed on the target tumor cell and a protein expressed by effector T-cells, resulting in their coupling. Commonly, those molecules, known as a Bispecific Antibodies (BsAbs), recruit and activate T-cells through binding a CD3 subunit of the T cell receptor complex for redirected lysis of tumor cells. This approach has been used to redirect T-cells toward a variety of TAAs, including CD19, Her2, and CD20 targets. Perhaps the most advanced BsAb is Blinatumomab [39], an investigational Bispecific T cell engager (BiTE®) antibody construct designed to direct T-cells against target cells expressing CD19 expressing B-cell derived leukemias and lymphomas, which has received orphan drug designation from the FDA for a wide array of CD19 expressing hematological malignancies. While BsAbs have shown promising results in liquid tumors and in preclinical studies of solid tumors, their clinical activity has been limited in solid malignancy but may be enhanced with more robust activation of effector T-cells.

Here, we sought to establish proof of concept for a unique approach that combines the advantages of BsAb therapies and gene-modified T-cell therapy. To address the issue of insufficient lymphocyte activation mediated by BsAbs, we designed, constructed and functionally characterized genetically-modified human T-cells expressing a unique co-stimulated immunoreceptor for bispecific antibody engagement, referred to as a BsAb-IR. Analogous to conventional BsAbs, our novel platform allows for the redirection of both CD8+ and CD4+ T-cells expressing a BsAb-IR against tumor targets via interaction with unique frBsAb intermediate, however, incorporation of a costimulatory signaling domain within the BsAb-IR construct results in heightened T-cell function. frBsAb-mediated activation of costimulated BsAb-IR-28z T-cells elicited enhanced production of multiple Th1-type proinflammatory cytokines, including IFN-g, TNF-a and IL-2, in comparison to activated BsAb-IR-z T-cells that lack a costimulatory signaling endodomain. These results are consistent with the notion that BsAb-IR T-cell have the capacity to receive simultaneous TCR and costimulatory signals via crosslinking of BsAb-IRs on the T-cell surface upon frBsAb engagement. In addition to cytokine secretion, antigen-specific target cell lysis occurs when BsAb-IR T-cells are redirected by TAA-specific frBsAb against TAA-expressing tumor cells. To the best of our knowledge, this strategy of utilizing primary human T-cells engineered to express a co-stimulated immune receptor for BsAb-mediated anti-tumor activity has not been previously reported. Although the specific tumor lysis mediated by this first generation of redirected BsAb-IR T-cells was less robust than previously reported for T-cells redirected with conventional BsAb [30,40], this platform is highly tunable for optimization. One likely explanation for this modest lytic activity is the low concentration of functionalized frBsAb heterodimer used in these assays, which was less than 20% of total antibodies mixture following heteroconjugation. Accordingly, selective isolation of functional heterodimers from the antibody mixture may significantly improve in vitro lytic activity of BsAb-IR T-cells.

Activation of T-cells is triggered by signals transmitted through the T-cell receptor/signal transduction complex (TCR/CD3 signal 1). Other costimulatory receptor-ligand interactions between T-cells are needed, however, for comprehensive T-cell stimulation (signal 2). One of the best-characterized costimulatory molecules is CD28, which plays a complex role in regulating T-cell function after interaction with its ligands, B7-1 (CD80) and B7-2 (CD86) [41,42]. Costimulation through CD28 and TCR/CD3 complex leads to high-level production of IL-2 and regulates T-cell proliferation [33]. Moreover, CD28 costimulation is thought to be required not only to activate T-cells but also to protect them from apoptosis mediated by the CD95 (APO-1/Fas) death receptor [43]. As reported, the lack or loss of CD28 ligands expression by tumors may, therefore, lead to insufficient T cell activation [44,45], where continued signaling through the T-cell receptor alone (CD3) can lead to anergy or antigen induced cell death (AICD) [46,47]. Thus, T-cells redirected and activated by a conventional CD3-signaling BsAb bound to the cancer cell surface are unlikely to obtain necessary secondary costimulatory signals, and may result in inefficient BsAb-mediated T cell activation and antitumor activity in patients. In previous studies, co-administering anti-CD28 antibodies or B7-scFv fusion proteins was shown to deliver a costimulatory signals [48,49]. However, this kind of systemic activation of the costimulatory ligand was accompanied by unexpected systemic toxicities *in vivo*, as observed after superagonistic anti-CD28 mAb TGN1412 application [50,51]. For this reason, and in order to localize the immunologic response to the tumor, others have attempted to transfer costimulatory ligands into the tumor cells. Indeed, 4-1BBL expression by tumor cells inhibited tumor outgrowth and induced strong cytotoxic T-cell response followed by long-term immunity in diverse mouse models [52]. However, the clinical applicability of such a strategy is challenged by the need for selective, efficient and safe gene transfer into the tumor cells. Thus, the generation of a novel, costimulated and specific receptor composed of a short binding moiety linked to T-cell signaling domains, CD3z and CD28, and construction of a novel bispecific antibody against a TAA and the extracellular domain of the costimulatory immune-receptor, may circumvent some of the potential major limitations associated with infusing co-stimulatory ligands together with BsAbs, specifically by permitting simultaneous activation and costimulation of a pre-selected T cell population in localized antigen-rich environment, As a proof of concept, we chose the extracellular portion of folate receptor alpha (FRa) as a BsAB partner. FRa is a 38 kDa glycosylphosphatidylinisotol (GPI)-anchored protein that binds folic acid with high affinity and transports folate by receptor-mediated endocytosis [53]. FRa exhibits limited normal tissue distribution, with measurable expression restricted largely to the apical surfaces of a few epithelia, predominantly in the kidney cortex, lung and choroid plexus [54]. Restricted distribution, and importantly, the lack of FRa expression on T-cells, makes this antigen a good candidate for a creation of a unique T-cell surface CD28 costimulated engager for bispecific antibody, the frBsAb-IR, yet other binding partners are under investigation.

In these principle-establishing studies, we generated bispecific antibodies by chemical conjugation (Additional file 1: Figure S1) and demonstrated that these hybrid molecules can redirect specific cytotoxicity of BsAb-IR engineered T-cells toward tumor cells expressing CD20 in vitro. These BsAbs were comprised of clinical-grade bivalent antibodies with low heterodimerism. While bivalency may increase IR crosslinking, limited heterodimers may reduce overall potency. To address this, future studies that incorporate newer BsAb formats, such as BiTE (bi-specific T cell engager) [55] and DART (dual affinity retargeting) [56], which have proven to be more potent than chemically conjugated BsAbs, are warranted.

In line with the known role of costimulatory signals in T-cell function [33], we found that BsAb-IR-28z T-cells produced at least three Th1 type cytokines, IFN-g, IL-2 and TNF-a, as well as MIP-1a, at levels that were significantly higher than those produced by BsAb-IR-z T-cells, following the engagement by frBsAb. This is of special importance, since the release of multiple cytokines and chemokines may recruit and activate endogenous immune cells to enhance the local antitumor response. Taken together, our in vitro data illustrate the high potential of a costimulated BsAb-IR approach, and supports its further optimization and evaluation in preclinical models. Our in vitro findings are in line with previously published data showing an enhanced BsAb-mediated antitumor response by provision of a CD137 signal in trans using a second recombinant Ab directed against a non-tumor specific cellular target [57]. In contrast to this approach, BsAb-IR has the unique advantage to simultaneously and locally activates selected population of gene engineered T-cells, and trigger the co-stimulatory signals upon cross-linkage of the BsAb-IRs to its target cell by application of one single bispecific molecule.

## Conclusions

In conclusion, a strategy of using genetically modified primary human T-cells expressing a co-stimulated immune receptor and armed with bispecific molecules offers the possibility of a straightforward method for delivery of co-stimulatory signals to antigen-redirected cytotoxic T-cells. This approach extends upon our experience in bridging tumor cells with T-cells using biotinylated antibodies that engage an avidin-based immunoreceptor on the engineered T-cell surface [58], however, by comparison, the BsAb-IR described here is fully human in composition reducing the risk for transgene immunogenicity, and can be bound with antibodies with affinities higher than that obtainable using dimeric avidin binding to biotin (KD = 0.5 +/− 0.1 nM). BsAb-IR T-cells encountering antigen-bound frBsAbs undergo potent activation that is enhanced by coordinate costimulatory signaling stemming from the BsAb-IR itself. Since CD28 costimulatory signals may not only bolster augmented T-cell function but also prevent T-cell AICD, we believe that the principles established in this study will significantly enhance anti-tumor activity of targeted BsAb-based tumor immunotherapy.

## Additional files

Additional file 1: Figure S1. Schematic illustration of the construction of anti-BsAb-IR × TAA bispecific antibody (frBsAb). Anti-BsAb-IR (MOV18 Ab) is cross-linked with Traut’s reagent and anti-TAA is cross-linked with SulfoSMCC [sulfosuccinimidyl 4-(N-maleimidomethyl) cyclohexane-1-carboxylate; before heteroconjugation overnight under the conditions described. Additional file 2: Figure S2. Tumor killing by redirected BsAb-IR T-cells is antigen-specific. CD20frBsAb redirected cytotoxicity of the CD20-negative AE17 cell line by BsAb-IR T-cells was not observed. Primary human T-cells transduced to express BsAb-IR-28z or GFP (control) were co-cultured with Cr^51^-labeled CD20-negative AE17, mouse mesothelioma cell line, pretargeted with indicated frBsAb for 4 hrs at the indicated effector to target ratio. Percent specific target cell lysis was calculated as (experimental - spontaneous release) ÷ (maximal - spontaneous release) × 100. Data represent the means ± SD for 3 different experiments.

## Abbreviations

TCR: T-cell Receptor
IFNg: Interferon gamma
IL2: Interleukin 2
GM-CSF: Granlulocyte-macrophage colony-stimulating protein
TNFa: Tumor necrosid factor alpha
MIP-1a: Macrophage inhibitory protein
BsAb-IR, TAA: Tumor Associated Antigen
CAR: Chimeric Antigen Receptor
BsAb: Bispecific antibody
