# Supplementary figures and images for: Targeted cancer immunotherapy via combination of designer bispecific antibody and novel gene-engineered T cells
Source: J Transl Med. 2014 Dec 13;12:347. doi: 10.1186/s12967-014-0347-2 (PMC4272781; doi:10.1186/s12967-014-0347-2)

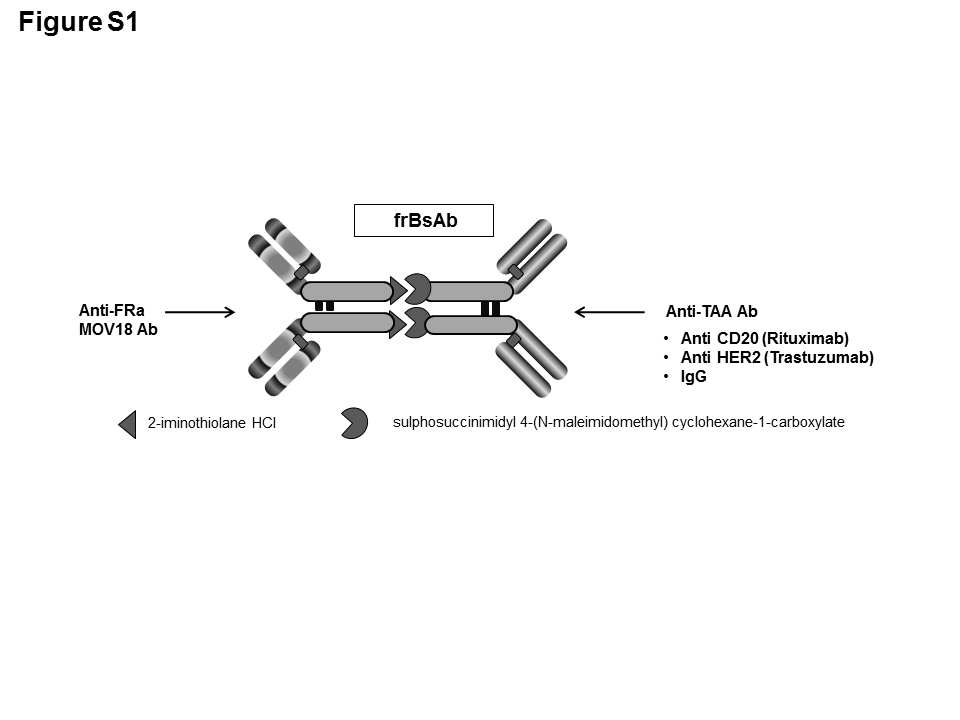

Supplement: Additional file 1: Figure S1. — Schematic illustration of the construction of anti-BsAb-IR × TAA bispecific antibody (frBsAb). Anti-BsAb-IR (MOV18 Ab) is cross-linked with Traut’s reagent and anti-TAA is cross-linked with SulfoSMCC [sulfosuccinimidyl 4-(N-maleimidomethyl) cyclohexane-1-carboxylate; before heteroconjugation overnight under the conditions described. [file 12967_2014_347_MOESM1_ESM.tiff]

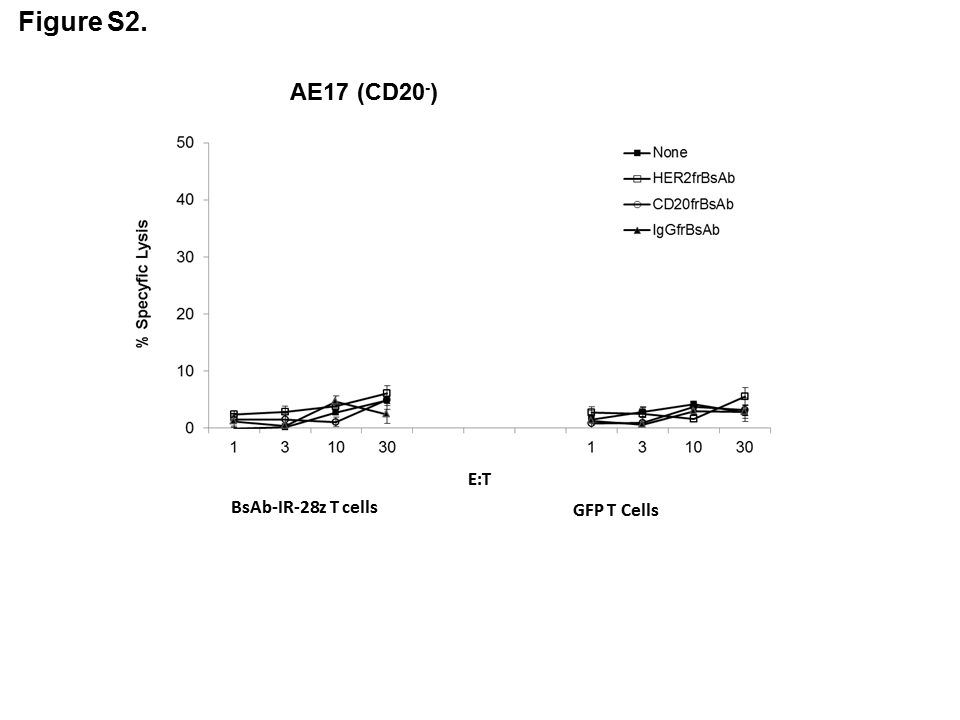

Supplement: Additional file 2: Figure S2. — Tumor killing by redirected BsAb-IR T-cells is antigen-specific. CD20frBsAb redirected cytotoxicity of the CD20-negative AE17 cell line by BsAb-IR T-cells was not observed. Primary human T-cells transduced to express BsAb-IR-28z or GFP (control) were co-cultured with Cr51-labeled CD20-negative AE17, mouse mesothelioma cell line, pretargeted with indicated frBsAb for 4 hrs at the indicated effector to target ratio. Percent specific target cell lysis was calculated as (experimental - spontaneous release) ÷ (maximal - spontaneous release) × 100. Data represent the means ± SD for 3 different experiments. [file 12967_2014_347_MOESM2_ESM.tiff]
